# Post infectious fatigue and circadian rhythm disruption in long-COVID and other infections: a need for further research

**DOI:** 10.1016/j.eclinm.2025.103073

**Published:** 2025-01-18

**Authors:** Achilleas Livieratos, Steven W. Lockley, Sotirios Tsiodras

**Affiliations:** a Independent Researcher, Athens 152 38, Greece; b Surrey Sleep Research Centre, School of Biosciences, University of Surrey, Surrey, GU2 7YW, UK; c 4th Department of Internal Medicine, Attikon University Hospital, Athens 124 62, Greece

**Keywords:** Long COVID, Post-infectious fatigue, Circadian rhythms, Chronic fatigue syndrome

## Abstract

Chronic fatigue syndrome (CFS) remains a subject of scientific research specifically with regards to its association with infections, including the more recently described Long COVID condition. Chronic fatigue and sleep disturbances in Long COVID are intricately linked to disruptions in circadian rhythms, driven by distinct molecular and cellular mechanisms triggered by SARS-CoV-2 infection. This can be driven by various mechanisms including dysregulation of key clock genes (CLOCK, BMAL1, PER2), mitochondrial dysfunction impairing oxidative phosphorylation, and cytokine-induced neuroinflammation (e.g., interleukin-6, tumor necrosis factor-alpha). Epigenetic changes, including DNA methylation at clock-related loci, particularly in peripheral tissues, further contribute to systemic circadian dysregulation. This work underscores the multifaceted molecular and systemic disruptions to circadian regulation in relation to fatigue and sleep disturbances identified as post-infectious sequelae, focusing on the Long COVID condition.

## Introduction

Fatigue, both general and chronic, significantly disrupts daily life and is a prominent feature of various chronic conditions, including post-infectious syndromes.^1^ CFS, also referred to as myalgic encephalomyelitis (ME/CFS), is characterized by persistent, debilitating tiredness lasting at least six months that is not alleviated by rest and is unrelated to excessive physical activity.^2^^,^^3^ Patients often report hallmark symptoms such as post-exertional malaise and unrefreshing sleep, with non-restorative sleep being a key diagnostic criterion.^2^, ^3^, ^4^

Emerging evidence suggests that disrupted circadian rhythms and altered cortisol levels may play a critical role in these conditions, contributing to sleep fragmentation, cognitive impairments, and dysautonomia.^5^, ^6^, ^7^

The interplay between infection, immune responses, and circadian disruption remains poorly understood but appears central to the pathophysiology of fatigue syndromes.^8^^,^^9^

This paper focuses on unraveling this complex relationship, with particular attention to Long COVID as a model for understanding post-infectious fatigue syndromes. By highlighting circadian dysregulation as a potential therapeutic target, we aim to underscore the importance of further research in this area and propose pathways for advancing clinical understanding and interventions.

## CFS, sleep and circadian rhythm dysregulation, and association with infection

### Fatigue epidemiology

General fatigue, defined as fatigue lasting less than six months, affects 24.2% of the global population, while chronic fatigue, persisting for more than six months, has a prevalence of 7.7%.^1^ CFS or CFS-like conditions are less common, affecting approximately 1.2% of the population.^1^ Among adults aged 18 and older, 20.4% report general fatigue, and 10.1% experience chronic fatigue, compared to 11.7% and 1.5%, respectively, in minors.^1^^,^^3^

Moderate fatigue is generally more common than severe fatigue, with an overall prevalence of 15% compared to 6.1%.^1^^,^^7^ Interestingly, in minors, severe fatigue surpasses moderate fatigue in prevalence (12.9% vs. 7.6%).^1^^,^^7^ Differentiating medically explained from unexplained fatigue is essential, as unexplained persistent fatigue, occurring without any underlying medical condition, is more prevalent (4.1% vs. 1.5%).^1^^,^^7^ Among adults, unexplained fatigue is 3.3 times more common than explained fatigue.^1^^,^^7^

### Chronic fatigue, infections and other entities

Chronic fatigue encompasses a spectrum of conditions, including ME/CFS, that are often associated with infectious diseases. For example, post-treatment Lyme disease syndrome and ME/CFS share similarities in prolonged fatigue, activity impairment, and disrupted sleep.^10^ Other infections, such as those leading to long-term symptoms e.g., the long-COVID-19 condition, suggest shared pathophysiological mechanisms (Table 1).

**Table 1:** Representative studies of infectious diseases associated with CFS.

Study	Year	Design	N of observations	Period	Infections associated	Comments
Cai et al.^11^	2024	Retrospective cohort	135,161 infected 5,206,835 controls	2020–2023	SARS-CoV-2 infection	• Non-hospitalized, Fatigue incidence: 2.6 (95% CI: 0.1–5.2) Disability Adjusted Life Years (DALYs) per 1000 persons • Hospitalized, Fatigue incidence: 11.7 (95% CI: 0.2–23.2) DALYs per 1000 persons
Chang H et al.^12^	2023	Prospective cohort Cox proportional hazards regression analysis	395,811	2000–2017	• *Mycobacterium tuberculosis* • Candida • *Staphylococcus aureus* • Salmonella • Influenza • *Escherichia coli* • Varicella-zoster virus	• Incidence density 5.40 per a thousand person-years • Hazard Ratio (HR): 1.5 (95% CI: 1.47–1.54)
Hsu HJ et al.^13^	2024	Nested case-control Cox-proportional hazards	2 million	2000–2017	• Pseudomonas • *Klebsiella pneumoniae* • *Haemophilus influenzae* • *Streptococcus pneumoniae* • Influenza	• Excluded < 20 yrs of age • HR: 1.5 (95% CI: 1.32–1.5)
Cornelissen MEB et al.^14^	2024	Multicentre prospective observational	91	2021–2022	SARS-CoV-2 infection	• 75.9%, at 3–6 months post-infection • 57.1%, 9–12 months post-infection
Bonilla H et al.^15^	2023	Retrospective cohort	140	2021–2022	SARS-CoV-2 infection	• 1/3 of patients had a severe functional decline • 43% ME/CFS
Schmidbauer L et al.^16^	2023	Observational study	425	2020–2021	SARS-CoV-2 infection	• 37% developed Post-COVID Fatigue
Walker S et al.^17^	2023	Cross-sectional observational	3754	2020–2022	SARS-CoV-2 infection	• 51% lost more than one day from work • 20% unable to work
Bretherick AD et al.^18^	2023	Population-based	17,074	2022	• Glandular fever • SARS-CoV-2 • Other • No infection	• Glandular fever (17%) • SARS-CoV-2 (2%) • Other infection (44%) • No infection at onset (16%) • Unsure (21%)
Hickie I et al.^19^	2006	Prospective cohort	253	–	• *Coxiella burnetii* (Q fever) • Glandular fever • Ross River virus	• 11% met criteria for chronic fatigue syndrome • Occurred at a similar incidence after each infection
Mørch K et al.^20^	2013	Translational research design	53	2007–2009	Giardia duodenalis	• CFS was diagnosed in 41.5%
Merikanto I et al.^21^	2023	International survey study	13,628	2021	SARS-CoV-2	Long-lasting symptoms prevalent among severe cases: • Fatigue: 61.3% • Insomnia: 49.6% • Excessive daytime sleepiness: 35.8%

Chronic fatigue is also a hallmark of diseases like systemic lupus erythematosus (SLE), multiple sclerosis (MS), rheumatoid arthritis (RA), post-polio syndrome (PPS), and type 1 diabetes (T1D).^8^ Fatigue prevalence rates are significant, ranging from 40% to 90% in these conditions, often leading to profound lifestyle impairment.^8^ Cancer-related fatigue is another critical example, frequently observed in advanced malignancies due to cachexia, metastases, and treatment side effects.^22^^,^^23^

These overlapping fatigue syndromes underscore the potential shared physiological mechanisms, such as systemic inflammation and circadian disruption, that may also link post-infectious states with ME/CFS. Similarly, conditions like fibromyalgia and irritable bowel syndrome highlight potential relevance, though their relationship to circadian dysregulation and post-infectious states requires further exploration. Notably, these chronic conditions are not classified as post-infectious syndromes, and their interaction with concurrent infections remains under-researched.

## Sleep and circadian rhythm dysregulation and association with fatigue

### Basic physiology of the circadian cycle

Circadian rhythms are daily physiological, metabolic, and behavioral cycles regulated by an internal system of clocks.^24^ The central clock, located in the suprachiasmatic nucleus (SCN), is primarily synchronized to light-dark cycles, coordinating peripheral clocks in various organs to maintain systemic harmony.^24^ These rhythms evolved to anticipate environmental changes, such as infections, with evidence showing that infection susceptibility varies with circadian timing.^24^^,^^25^

Circadian disruption can occur due to misalignment between central and peripheral clocks, external time cues, or reduced amplitude of physiological rhythms.^26^ Misaligned sleep, such as delayed or fragmented patterns, often indicates circadian disturbances but requires objective markers to confirm.^26^ Conditions like delayed sleep phase syndrome highlight this misalignment.^27^^,^^28^ Clinical assessments, including patient-reported outcomes, can help identify and address potential circadian rhythm deregulation in affected individuals.^29^

### Evaluation of sleep disruption

Sleep-wake patterns and their alignment with circadian rhythms can be assessed using tools like sleep logs or actigraphy.^30^ However, translating these measures into accurate circadian data is challenging due to inter-individual variability and masking effects from external factors like light and activity.^31^, ^32^, ^33^, ^34^, ^35^, ^36^ Standard biomarkers, such as melatonin (dim light melatonin onset or DLMO) and cortisol levels, remain the gold standard for measuring circadian time under controlled conditions.^37^^,^^38^ Advances in molecular techniques, including clock gene expression studies, show promise but require further validation.^39^, ^40^, ^41^

### Clinical evidence linking fatigue with sleep disturbance

Sleep disturbances are closely linked to fatigue across chronic conditions, including MS, SLE, and ME/CFS.^9^ Poor sleep quality and irregular patterns exacerbate fatigue severity, as observed in MS and SLE patients, where nocturnal melatonin reductions correlate with disease severity.^8^^,^^42^ Sleep-related fatigue differs from chronic fatigue driven by broader physiological factors like inflammation and neurological changes.^8^

ME/CFS patients exhibit distinct sleep and circadian abnormalities, such as reduced light exposure, irregular activity patterns, and delayed melatonin timing.^43^, ^44^, ^45^, ^46^, ^47^ Studies have highlighted disruptions in circadian rhythms, including delayed DLMO, which may contribute to fatigue and other symptoms.^46^ These findings underscore the need for further research into circadian biomarkers and their role in ME/CFS.

### Potential interventions for circadian rhythm regulation and association with fatigue and CFS/ME

Circadian rhythm interventions, such as light-dark exposure and melatonin supplementation, show promise in mitigating fatigue. Synthetic melatonin, particularly when administered in alignment with individual DLMO, has demonstrated significant improvements in fatigue severity and cognitive function among ME/CFS patients with delayed sleep-wake phase disorder (DSWPD).^46^^,^^47^ Although the exact mechanisms remain unclear, melatonin’s ability to phase-shift circadian rhythms may contribute to these benefits.^46^

Melatonin and zinc supplementation have shown systemic benefits for chronic fatigue patients, though effects on sleep quality and mood remain inconclusive.^47^^,^^48^ Additionally, melatonin’s anti-inflammatory properties may reduce symptoms associated with infectious diseases and restore circadian balance in specific contexts, such as intensive care, though evidence remains limited.^48^^,^^49^

## Infection-associated fatigue and sleep and circadian rhythm dysregulation - the long COVID example

### Overview of long COVID and fatigue

Long COVID, also termed post-COVID-19 condition, is characterized by persistent symptoms lasting at least three months after SARS-CoV-2 infection, with fatigue being a predominant manifestation.^50^ This condition represents one of the most recently characterized examples of post-infectious fatigue syndromes.

### Long COVID epidemiology

Long COVID affects a significant proportion of SARS-CoV-2-infected individuals, with severity often linked to the initial disease course. Hospitalized patients experience a markedly higher incidence of disability and increased mortality risk than non-hospitalized patients.^11^ Non-hospitalized individuals showed milder disability impacts, though fatigue remained a common and persistent symptom.^11^

Recent studies suggest that Long COVID symptoms, including fatigue, are similar to those reported after other viral infections like influenza.^51^^,^^52^ In both SARS-CoV-2 and influenza cases, approximately 16% of individuals report ongoing symptoms one year post-infection, with 3.6% experiencing moderate-to-severe functional impairment.^51^ This underscores the broader relevance of post-viral fatigue syndromes.

### Long COVID and chronic fatigue syndrome mechanistic overlap

Long COVID shares significant pathophysiological overlap with ME/CFS, particularly in systemic inflammation, neuroglial dysfunction, and mitochondrial impairment.^43^^,^^44^ Elevated pro-inflammatory cytokines (e.g., IL-6, TNF-α) and neuroinflammation are common in both conditions, contributing to sustained fatigue and cognitive dysfunction.^53^^,^^54^ Mitochondrial dysfunction and metabolic reprogramming exacerbate energy deficits, while circadian rhythm disruptions are prevalent.^54^^,^^55^

Long COVID patients often exhibit ME/CFS-like sleep and circadian rhythm disturbances, including fragmented sleep, diminished motor activity, and disrupted temperature rhythms.^54^, ^55^, ^56^, ^57^ Actigraphy studies reveal reduced inter-daily stability and increased intra-daily variability, reflecting impaired circadian regulation likely driven by persistent inflammation and mitochondrial dysfunction.^54^, ^55^, ^56^, ^57^, ^58^

### Sleep and circadian rhythm dysregulation and association with post-infectious fatigue in long COVID

Pre-existing sleep disturbances, such as insomnia and obstructive sleep apnea (OSA), significantly increase the risk of developing Long COVID.^56^^,^^59^ Severe initial infections, particularly those requiring hospitalization or ICU care, are associated with more pronounced sleep and circadian disruptions.^54^, ^55^, ^56^ Up to 70% of ICU survivors report persistent sleep disturbances, including reduced slow-wave sleep and fragmented REM patterns, often linked to systemic inflammation and ICU stressors.^54^, ^55^, ^56^

Regarding circadian deregulation, shift work, an example of misalignment between circadian time and behavior is correlated with increased risk of in-hospital SARS-CoV-2 infectivity.^60^ Such risk may be averted, or at least minimized, via immunization of high-risk patients.^61^^,^^62^ There is also some evidence that vaccination responses could be improved by inoculating individuals at a more optimal circadian time.^63^ The outcome could be affected even long after the initial infection and the association likely depends on the type and tropism of the virus and other factors like the age of the host.^24^

In non-hospitalized patients, sleep disturbances, while less severe, remain prevalent. Insomnia, hypersomnia, and unrefreshing sleep affect 35–40% of individuals, with symptoms often persisting for months post-infection.^54^, ^55^, ^56^ Longitudinal studies have found that individuals with probable Long COVID in this group have 2.6 times higher odds of developing new sleep disturbances, such as difficulty initiating sleep, nighttime awakenings, or excessive daytime sleepiness, compared to pre-infection baseline.^54^, ^55^, ^56^ These disturbances underscore the widespread impact of SARS-CoV-2 on sleep dynamics, necessitating targeted evaluation and intervention strategies to address the distinct needs of hospitalized and non-hospitalized Long COVID populations.^54^, ^55^, ^56^

A recent study also explored the possible connection between circadian rhythm disruptions and Long COVID, drawing comparisons to similar disruptions seen in ME/CFS.^64^ The study surveyed 314 adults, that were in the past 3 months infected by SARS-CoV-2, identifying a “Long COVID” group based on ongoing symptoms.^64^ Most of this group reported new symptoms, including fatigue (around 80%) and attention deficits (around 70%), along with significant difficulties in falling asleep and waking up at desired times.^64^ These sleep timing issues could be indicative of a circadian delay (DSWPD), although further confirmation is required.^64^ The mechanisms by which immune and inflammatory responses triggered by viral infections like SARS-CoV-2 may contribute to circadian rhythm disturbances, which could lead to further deterioration and emergence of fatigue, cognitive dysfunction, and sleep issues in Long COVID patients, remains to be fully elucidated in future research.

While previous literature has established an association between sleep disruption and host response in infection, chronic sleep deprivation, clock-disrupted individuals, including shift workers, or patients with underlying circadian disorders are potentially at an increased risk of infection.^65^, ^66^, ^67^, ^68^ Regular or permanent shift work and irregular or permanent night shifts have been correlated with SARS-CoV-2 positivity vs. controls.^60^ Another study also reported that 40% of its study population, including over 30% of the morning and around 17% of the evening chronotypes, were infected with SARS-CoV-2.^65^ Sleep disturbance can additionally be a sequela of infection and has been commonly described as a feature of the post-COVID-19 condition with approximately 10–20% of individuals reporting such a symptom and the associated prevalence of sleep problems varying significantly, from as low as 6% to over 70%.^66^ An international online survey showed that 78.6% of participants with post-COVID-19 reported disruptions in their sleep, such as sleep fragmentation, delayed sleep onset, and circadian rhythm sleep-wake abnormalities.^66^ Sleep pattern irregularities often manifest between two weeks and up to a year or more after hospitalization or testing for SARS-CoV-2.^66^ Female patients under 50 years old who experienced severe SARS-CoV-2 infections reported poorer outcomes.^66^

### Long COVID: mechanistic insights and therapeutic implications

Circadian rhythm disruptions in Long COVID are likely influenced by immune dysregulation, autonomic dysfunction, and mitochondrial impairment.^54^, ^55^, ^56^, ^57^, ^58^, ^59^, ^60^, ^61^, ^62^, ^63^, ^64^, ^65^, ^66^ Elevated cytokines disrupt hypothalamic control of melatonin synthesis, compounding sleep disturbances and perpetuating fatigue.^54^, ^55^, ^56^, ^57^ Autoantibodies targeting β-adrenergic and muscarinic receptors further disrupt autonomic and circadian processes, exacerbating symptoms.^69^

In a case control study with healthy volunteers ME/CFS affected approximately 10–12% of individuals following certain infections, with a notable association with Epstein–Barr Virus and potentially SARS-CoV-2.^70^ A critical finding was the distinct biological differences between male and female ME/CFS participants.^69^ Gene expression analyses revealed that less than 5% of differentially expressed genes overlapped between sexes, indicating significant sex-specific pathways involved in the disease.^70^ For instance, males exhibited upregulation in T-cell activation and proteasome pathways, while females showed changes in B-cell proliferation processes.^70^ On the other hand, circadian and sleep disruptions were notable in the post-infectious ME/CFS (PI-ME/CFS) cohort.^70^ Polysomnography revealed no clinically relevant differences in sleep architecture between PI-ME/CFS patients and healthy volunteers.^70^ However, autonomic dysfunction was evident, with diminished heart rate variability and altered sleep patterns suggesting increased sympathetic and decreased parasympathetic activity.^70^ These findings highlight the significant impact of rhythm disturbances may have on the overall fatigue experienced by patients after an infection.^70^ Interestingly, post-COVID-19 associated symptoms have been corelated with reduced levels of serotonin.^71^ This may arise from reduced tryptophan intestinal absorption, following an interferon-driven inflammatory response to viral infection.^71^ This in turn may impact hippocampal brain activity and form the basis of a mechanistic link between gastrointestinal absorption mechanisms and neurocognitive impairment and dysfunction following an acute infection especially one that involves the gastrointestinal tract.^71^

Moreover, SARS-CoV-2 induces metabolic reprogramming and epigenetic modifications that disrupt circadian regulation. Mitochondrial dysfunction reduces ATP availability, impairing the circadian clock machinery.^72^, ^73^, ^74^ Epigenetic alterations, such as DNA methylation changes at circadian gene loci, perpetuate systemic misalignment.^72^, ^73^, ^74^ Moreover, the virus's ability to affect oxidative phosphorylation impacts not only cellular metabolism but also the signaling pathways that coordinate energy-dependent processes like sleep-wake cycles.^72^, ^73^, ^74^ Elevated pro-inflammatory cytokines (IL-6, TNF-α) interfere with hypothalamic control of melatonin synthesis, compounding sleep disturbances.^72^, ^73^, ^74^ These mechanisms are corroborated by studies showing mitochondrial transcriptomic changes.^72^, ^73^, ^74^ Melatonin may mitigate mitochondrial dysfunction in Long COVID, improve cellular immunity and support antioxidant properties.^75^

The adaptive immune system modulates the response to SARS-CoV-2, with T and B cells being central to the immune defence and potential contributors to Long COVID pathogenesis. SARS-CoV-2 induces a dynamic activation of CD4+ and CD8+ T cells, which mediate viral clearance through the secretion of interferon-gamma (IFN-γ) and direct cytotoxicity against infected cells. However, dysregulation of T-cell responses, characterized by excessive activation or exhaustion, has been observed in severe cases.^76^ Evidence of persistent B-cell activation and somatic hypermutation in germinal centers suggests that prolonged antigen exposure may contribute to immune dysregulation, potentially linking chronic immune activation to the symptoms of Long COVID.^76^

Persistent immune activation and cytokine release, hallmarks of Long COVID, can interfere with the hypothalamic-pituitary-adrenal (HPA) axis and alter normal signaling governing circadian homeostasis.^77^^,^^78^ For instance, elevated IL-6 and TNF-α during prolonged immune responses directly affect the SCN, the brain’s circadian pacemaker.^77^^,^^78^ These cytokines impair the SCN’s ability to regulate melatonin synthesis and secretion, which is crucial for sleep initiation and maintenance.^77^^,^^78^ In parallel, mitochondrial dysfunction driven by cytokine-induced oxidative stress exacerbates cellular energy deficits, which are tightly linked to circadian control over metabolic and physiological processes.^77^^,^^78^

Dysregulation of the autonomic nervous system (ANS) in Long COVID is mediated by autoantibodies targeting G-protein-coupled receptors (GPCRs), such as β-adrenergic and muscarinic receptors.^69^^,^^79^ Autoantibodies against β-adrenergic (β1 and β2) and muscarinic acetylcholine receptors (M3 and M4) have been detected at elevated levels in individuals experiencing Long COVID, with symptoms including chronic fatigue, sleep disturbances, and dysautonomia.^69^^,^^79^ These autoantibodies disrupt circadian processes, including melatonin secretion and energy metabolism, exacerbating symptoms like fatigue and sleep disturbances.^69^^,^^79^

### Long COVID and other Co-morbidities

Mood disturbances, including post-traumatic stress disorder (PTSD) and depression, exacerbate sleep-wake cycle disruptions through dysregulation of the HPA axis.^77^^,^^78^ Concurrent mood disturbances significantly exacerbate sleep disruptions in Long COVID patients, as psychological stress, anxiety, and depression interact with the physiological mechanisms underlying sleep regulation. Studies show that individuals recovering from COVID-19 who experience PTSD or depressive symptoms are more likely to report insomnia and poor sleep quality.^77^^,^^78^ For instance, a mediation analysis revealed that mental health factors such as PTSD directly influence the severity of insomnia in COVID-19 survivors, with higher PTSD scores correlating with worse sleep outcomes.^78^

With regards to the presence of other chronic conditions such as cardiovascular disease, chronic obstructive pulmonary disease, rheumatological conditions, malignancy and others one can only hypothesize that synergistic mechanisms exist to exacerbate fatigue, CFS/ME and Long COVID manifestations in these patients.

### Long COVID and potential role of viral evolution

The rapid evolution of SARS-CoV-2 has been characterized by mutations concentrated in key antigenic sites, especially within the spike (S) protein, necessary for viral invasion into host cells.^1^ Mutations in these antigenic sites contribute to immune evasion and prolonged viral persistence, exacerbating the risk of Long COVID.^80^ Notable mutations, such as E484K and N501Y, have been implicated in altering immune responses.^80^ Despite reduced virulence in later variants like Omicron (possibly additionally explained by the presence of hybrid immunity and/or previous immunization), their high transmissibility continues to include the possibility of a significant burden of post-acute sequelae.^4^^,^^76^ Genetic predisposition, including polymorphisms in genes regulating immunity and circadian rhythms, further modulates susceptibility to Long COVID and its severity.^4^^,^^76^^,^^80^

This mutational plasticity plays a significant role in the potential for Long COVID, as variants that evade the immune system might sustain prolonged viral presence in some individuals.^76^^,^^80^ Prolonged inflammation and viral persistence, especially in immunocompromised individuals, coupled with individual genetic predispositions, can amplify immune dysregulation, resulting in lingering symptoms that characterize Long COVID.^76^^,^^80^

### Potential interventions in long COVID associated with the circadian clock

Therapeutic interventions such as melatonin and zinc supplementation offer potential benefits.^75^ Melatonin's dual role as a chronobiotic and anti-inflammatory agent supports circadian alignment and mitigates mitochondrial dysfunction, while zinc aids immunity and clock gene function.^75^ On the other hand, apheresis have shown promise in reducing these autoantibody levels and improving clinical outcomes.^69^^,^^79^ Chronic inflammation and oxidative stress, hallmarks of Long COVID, further exacerbate ANS dysfunction, promoting fragmented sleep patterns and circadian misalignment.^69^^,^^79^

## Outstanding questions

### Sleep and circadian rhythms: clarifying misconceptions

A common misconception is equating sleep disruption directly with circadian rhythm disruption. While interlinked, these phenomena have distinct underlying mechanisms. Sleep disturbances may arise from pain, anxiety, or behavioral factors unrelated to circadian disorders, emphasizing the need for nuanced differentiation to avoid oversimplified conclusions and misdirected interventions.^26^

Accurate measurement of circadian markers is also a critical gap. Reliable markers, such as melatonin (dim light melatonin onset, DLMO), its metabolites, and cortisol, must be assessed under controlled conditions across a full 24-h cycle.^26^^,^^29^^,^^81^ Incomplete or single-point sampling often leads to misleading conclusions about circadian profiles.^26^^,^^29^^,^^81^

### Future research priorities

Future studies should stratify Long COVID patients based on factors like disease severity, pre-existing sleep disorders, and mood disturbances. Stratification may help identify subgroups with distinct pathophysiological mechanisms, enabling tailored interventions. Advanced tools, such as RNA sequencing of the blood transcriptome, could help map molecular disruptors associated with circadian misalignment and refine therapeutic approaches.^39^

There is also growing evidence of genetic predispositions influencing circadian rhythms and fatigue in ME/CFS and Long COVID patients. For instance, studies have identified the CLOCK gene as significantly correlated with circadian regulation and fatigue.^82^ Recognizing individual variability in circadian patterns is critical to understanding the heterogeneity of these conditions and tailoring interventions.

### Biomarkers and neuroimaging

The identification of novel biomarkers remains a high priority.^83^, ^84^, ^85^ Transforming growth factor beta (TGFB) has emerged as a promising candidate due to its influence on circadian rhythms and transcriptomic regulation.^83^^,^^85^ Neuroimaging studies have also revealed structural alterations in brain regions associated with sleep and cognition, such as the hippocampus and precuneus. These findings suggest that biomarkers, including TGFB, IL-6, TNF-α, and neuroimaging measures, could enhance our understanding of sleep disturbances and fatigue in Long COVID.^83^, ^84^, ^85^, ^86^

### Immunological dynamics and inflammation

The role of aging could be an additional important parameter including other factors like virus tropism.^87^ Inflammaging is a term describing the chronic, low-grade inflammation that develops with advanced age, that is closely related to immunosenescence, the slow decline of the immune system associated with aging through the reduction of the adaptive immune response.^87^ Both these processes may contribute to the increased susceptibility of older individuals to infection.^87^ This dysfunctional immune environment maybe related to chronic fatigue mediated by a pro-inflammatory cytokine milieu that could affect the central nervous system, potentially disrupting neurotransmitter signaling and leading to fatigue and cognitive impairments.^87^

On the other hand, exacerbated inflammatory responses in younger patients with infection due to specific pathogens may contribute to similar phenomena in younger individuals.^88^ This is often termed “cytokine storm” and can result in severe respiratory distress, multi-organ failure, and high mortality rates and potentially more severe post-infectious sequelae.^88^ The interaction between sleep disturbances and infection is underpinned by immune and inflammatory dynamics, with cytokines playing a central role.^83^^,^^89^ Sleep deprivation, for instance, has been shown to increase plasma levels of pro-inflammatory markers which contribute to systemic inflammation and disrupt normal sleep architecture.^83^^,^^89^ These cytokines exhibit pro-somnogenic properties, promoting slow-wave sleep in mild inflammation, but their excessive levels can suppress both non-REM (NREM) and REM sleep. Infections, such as SARS-CoV-2, further exacerbate this imbalance, due to increased inflammatory markers in Long COVID patients correlating with reported sleep disturbances.^83^^,^^89^

The interaction between immune dysregulation and circadian rhythms plays a pivotal role in post-infectious fatigue. Chronic inflammation, cytokine imbalances, and autonomic dysfunction exacerbate sleep disturbances and circadian misalignment.^87^, ^88^, ^89^ Addressing these dynamics through targeted therapies, may restore sleep integrity and mitigate systemic inflammation in affected patients.

### Host response and time-of-day vaccination

Emerging research suggests that circadian patterns influence the host response to immunization. Time-of-day vaccination may improve immune responses and could play a role in increasing vaccine acceptance.^63^ Further studies should explore the clinical advantages of circadian-aligned vaccination strategies.

### Conclusion and future directions

A critical interplay exists between infection, the host immune response, circadian rhythms, and the development of post-infectious fatigue. Increasing evidence links specific pathways of sleep and circadian disruption to infections and their sequelae, such as Long COVID and ME/CFS.

**Fig. 1:**
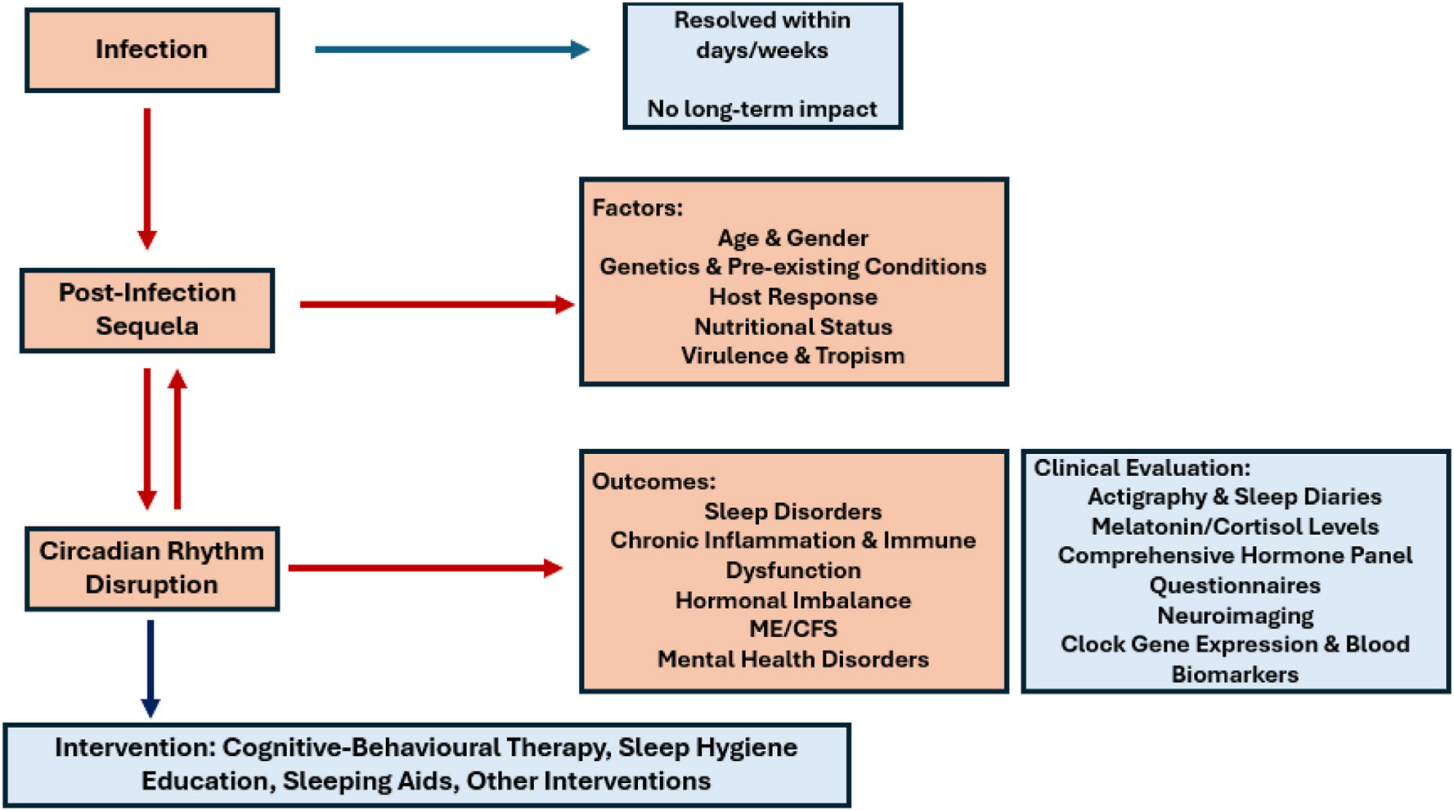
Schematic of interrelation of biological rhythms and infectious diseases.

**Fig. 2:**
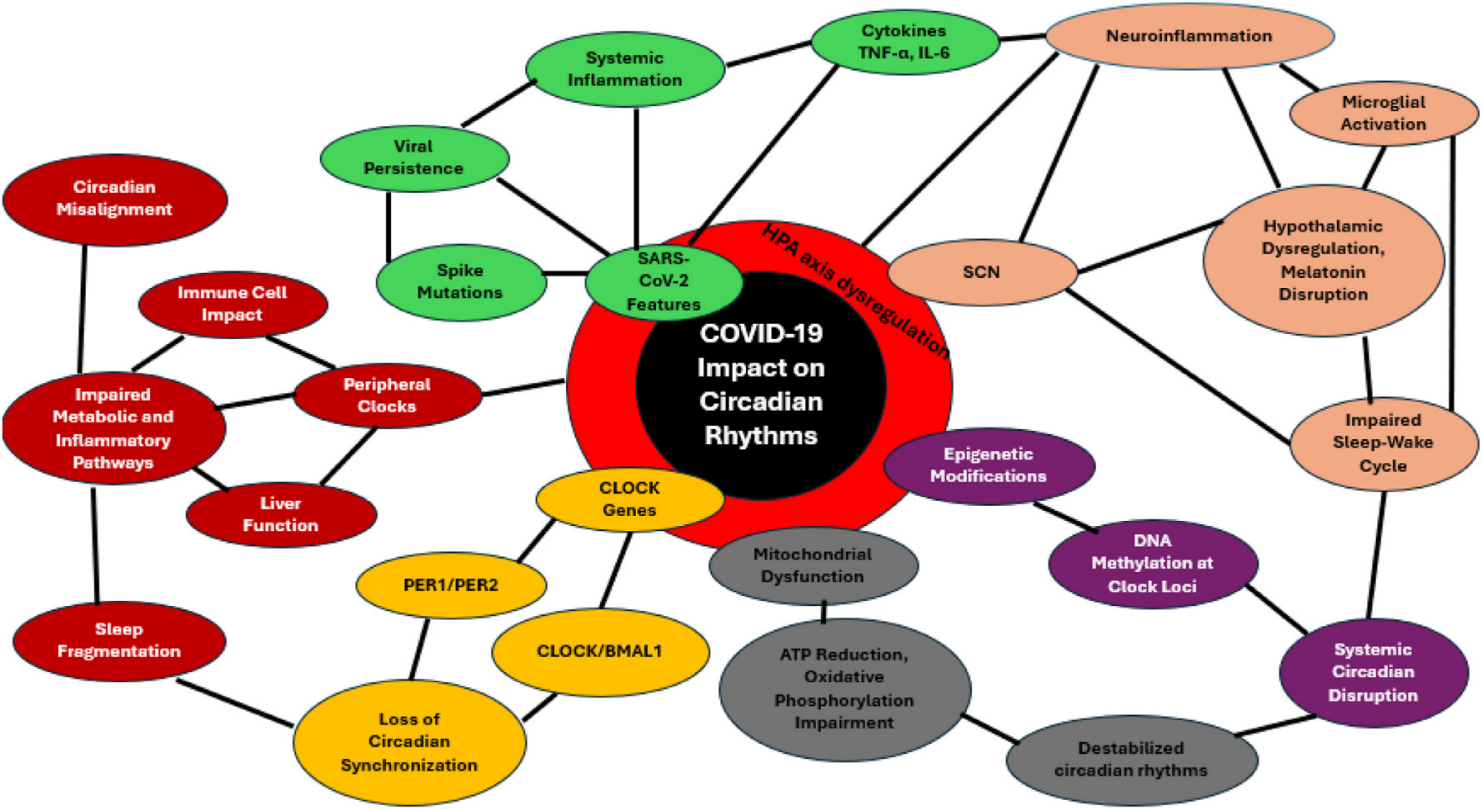
Diagram highlighting circadian disruption induced by COVID-19 and different mechanisms. HPA: Hypothalamic-Pituitary-Adrenal; SCN: Suprachiasmatic Nucleus.

We propose a schematic (Fig. 1) to illustrate the interconnected mechanisms contributing to these conditions and a second model (Fig. 2) detailing the impact of COVID-19 on circadian regulation. Sleep abnormalities, as prominent clinical findings, may also serve as quantifiable biomarkers for chronic fatigue conditions.

Future research should focus on unraveling the links between post-infectious fatigue-dominant syndromes, circadian dysregulation, and immune responses. Such studies could identify therapeutic targets for systemic conditions like ME/CFS and Long COVID, paving the way for more effective interventions and improved patient outcomes.

## Contributors

S.T. and A.L. conceived the idea; A.L., S.W.L and S.T. performed the literature search; A.L. and S.T. wrote the manuscript and drew the figures; S.W.L. and S.T. critically corrected the manuscript; S.T. oversaw the study. All authors have read and agreed to the published version of the manuscript.

## Data sharing statement

No new data were created or analyzed in this study. Data sharing is not applicable to this article.

## Declaration of interests

SWL has received consulting fees from Hintsa Performance, Light Cognitive, Lighting Science Group Corporation/HealthE, View, Ashurst Risk Advisory, and Monash University; has current consulting contracts with Apex 2100, KBR Wyle Services, Timeshifter, Illumalife and Absolute Rest; has received payment or honoraria for lectures, presentations, speakers bureaus, manuscript writing or educational events from Bloxhub/Lys, Danish Centre for Lighting, Clifton College, and CloserStill Media; and travel or accommodation expenses (no honoraria) from Wiley; has received royalties from Oxford University Press; previously held equity in Mental Workout, Akili Interactive, Light Cognitive, Consumer Sleep Solutions, HealthE and currently holds equity in iSleep, Timeshifter, and Illumalife; had a Clinical Research Support Agreement as principal investigator with Vanda Pharmaceuticals, two Project Grants as principal investigator with the National Police Wellbeing Service, a Project Grant as co-investigator with Transport for London, and currently has an Investigator-Initiated Trial (IIT) as co-investigator with Seoul Semiconductor, an IIT as co-investigator with BIOS, a sponsor-initiated trial as site-principal investigator with Vanda Pharmaceuticals, and a Development and Program Evaluation: Advanced Technologies (THFRAT) Solicitation as co-investigator with Volpe National Transportation Systems Center; is an unpaid Board Member of the Midwest Lighting Institute (non-profit); has served as a paid expert in legal proceedings related to light, sleep, and health; and has several patents related to circadian applications. All other authors declare no competing interests.
